# Effects of 8 weeks resistance training on plasma vaspin and lipid profile levels in adult men with type 2 diabetes

**Published:** 2014

**Authors:** Ali Barzegari, Hassan Amouzad Mahdirejei

**Affiliations:** 1 Physical education Department, Payam Noor University, 19395-4697, Babol, Iran.; 2 Department Of Exercise Physiology, Faculty Of Physical Education & Sport Science, Islamic Azad University, Sari Branch, Sari, Iran.

**Keywords:** Resistance training, vaspin, lipid profile, type 2 diabetes

## Abstract

***Background: ***Vaspin is associated with decreased blood glucose concentration. In this study, we aimed to investigate the effects of resistance exercise training on plasma vaspin and lipid profile levels in patients with type 2 diabetes.

***Methods:*** Thirty men were divided into 2 groups: control (n=15), and resistance exercise (n=15). The resistance group performed a resistance exercise consisting of 3 circuits of 8-15 repetitions of the 8 exercises. Lipid profiles and vaspin levels were measured at baseline and at the end of study. This study was registered in the Iranian Registry of Clinical Trial (www.irct.ir) with registration number ID: IRCT2013060911772N1.

***Results:*** Intergroup study showed that after an 8-week of resistance training, resistance group had reductions in vaspin (330.50±82.51 ng/ml vs 251.62±107.28 ng/ml, p=0.03), and TC levels (185.21±47.51 mg/dL vs 171.10±37.91 mg/dL p= 0.02); but, had increase in HDL-C levels (38.20±20.65 mg/dL vs 43.80±7.87 mg/dL p=0.01), whereas, vaspin levels significantly increased in control groups (344±78.64 ng/ml vs 436 ± 70.47 ng/ml, p= 0.03). On the other hand, significant difference was seen in plasma vaspin levels between two groups.

***Conclusion: ***Our findings suggest that resistance training significantly decreased the level of vaspin without affecting the lipid profile level.

Many clinical studies show that in an adult individual a decline in skeletal muscle mass has been termed sarcopenia. This loss of muscle mass increases in inflammatory cytokines and the risk of developing glucose intolerance as well as reduced lipid oxidative capacity (1). Hyperglycemia increases the risk of microvascular complications, while dyslipidemia is a major risk factor for macro vascular complications in patients with type 2 diabetes (T2D). Elevated low density lipoprotein (LDL)-cholesterol is a major risk factor for cardiovascular disease (2, 3). On the other hand, to investigate the link between adiposity and T2D, a variety of adipokines that modulate insulin sensitivity have been demonstrated (2). Vaspin (a visceral adipose tissue-derived serine protease inhibitor) is a novel adipocytokine that was reported to be specifically expressed in white adipose tissue (WAT) of Otsuka Long-Evans Tokushima Fatty (OLETF) rats, an experimental model for T2D and was postulated to have insulin sensitizing effects in state of obesity (2). Vaspin has been suggested as a compensatory factor against the insulin resistance state of metabolic syndrome (2). However, the relationship between vaspin and diabetes is still controversial (4). 

Oberbach et al. observed the significant reduction in serum vaspin concentrations after a 1-hour acute exercise bout as well as after 4 weeks of training in healthy young men and concluded that vaspin serum concentrations decreased by exercise-induced oxidative stress, but not by exercise associated improvement in insulin sensitivity (5).

However, the effects of lipid profile change induced by exercise training and resistance training (RT) on plasma vaspin concentrations in patients with T2D are still unclear. Thus, the aim of the present study was to investigate the effects of resistance exercise on vaspin and lipid profile levels in patients with type 2 diabetes.

## Methods

**Subjects: **In this quasi experimental study, thirty adult men with T2D participated in this study. The subjects were assigned to RT group (age 49.22±5.7 years, weight= 82.5±12.4 kg, BMI= 28.5±5.9 kg/m2, n=15) and control group (age 48.62±7.05 years, weight= 79.8±9.2, BMI= 27.3±4.3 kg/m2, n=15). The subjects were excluded if they had a known history of stroke or uncontrolled hypertension, severe dyslipidemia, or any other serious chronic disease requiring active treatment. Written informed consent was obtained from all the participants. To reduce drug effects, we selected the subjects with glycated hemoglobin values under 9% who received over 1,000 mg of metformin per day, and drug dosages were maintained throughout the study. In addition, pulse wave conduction velocity tests showed no significant differences between the two groups. Before initiating the tests, the participants underwent an anamnesis, a clinical evaluation and weight, height, body mass index and body fat mass measurements. Then all of them underwent familiarization sessions and participated in 1RM test. Before the 1RM test, the participants underwent familiarization session and familiarize themselves with the standard exercise techniques. The study protocol was approved by the local ethical committee and conducted in accordance with the Helsinki Declaration.

**Exercise Training Procedure: **The exercise group participated in 8 weeks (3 nonconsecutive days per week) of supervised circuit resistance exercise program. The programs composed of 3 steps: warm-up for 10-15 min, circuit resistance exercise, and cool down for 10 min. The resistance exercise program consisted of 8 isotonic exercises with 50-80% one-repetition maximum (1-rm) was performed in a circuit. During the ﬁrst and fourth weeks of training, the resistance was set at 50–70% of each individual’s 1RM with 8-15 repetitions of exercise movement within 45-60 seconds. Thereafter, the goal was to achieve between 70-80% of the current 1RM with 8-10 repetitions of exercise movement within 45-60 seconds. The participants performed 3 circuits of 8 exercises per training session. The intervals between each exercise were 30-60 seconds and between each circuit was 120-180 seconds. Ten exercises were used for the training: bench press, knee extension, knee flexion, lat pull-downs, seated rowing, bicep curls, heel raise, triceps extension exercises (6). 

**Research Design: **The anthropometric characteristics of the subjects are shown in table 1. The BMI (kg/m^2^) of each subject was calculated on the basis of their weight and height, and percentage body fat (PBF) was assessed using a bioelectrical impedance instrument (in-body -720, korea). Blood samples were collected in the morning after a 12-hour overnight fasting, before and after the 8-week of exercise program. Plasma vaspin levels were determined with vaspin enzyme-linked immunosorbent assay (ELISA) kit (Cusabio Biothech, Wuhan, China). Plasma total Triglyceride (TG) was determined by enzymatic colorimetric method by glycerol-3-phosphate oxidase (GPO) (Pars Azmoun, Tehran, Iran). Total cholesterol (TC) was determined by enzymatic photometric method using cholesterol oxidase-amino antipyrine (CHOD-PAP) (Pars Azmoun, Tehran, Iran). High-density lipoprotein cholesterol (HDL-C) was determined by direct colorimetric method (Randox, Antrim, UK), and the procedure of Friedewald et al. was used to estimate low-density lipoprotein cholesterol (LDL-C) (7). This study was registered in the Iranian Registry of Clinical Trial (www.irct.ir) with registration number ID: IRCT2013060911772N1

All data were expressed as mean ± standard deviations (SD) and analyzed using SPSS version 18.0. The differences between two groups were examined by a Co-Variance test, and the before and after comparisons within groups were performed using paired t-tests. The relation between variables was assessed using Pearson’s method. P-values below 0.05 were considered statistically significant. 

## Results

Participant characteristics at baseline can be seen in tables 1 and 2. There was no significant difference between the two groups at baseline in lipid profiles levels (TC, TG, HDL, LDL), and vaspin levels (table 2). The analysis of metabolic parameters, and vaspin level results showed that the mean of vaspin levels of subjects in the training group decreased from 330.50±82.51 to 251.62±107.28 ng/ml after 8 weeks of exercise, which was statistically significant, also, vaspin levels significantly increased in control groups (344±78.64 ng/ml vs 436±70.47 ng/ml). On the other hand, a decrease in vaspin levels showed significant difference between the two groups. The analysis of metabolic parameter results showed that the mean of cholesterol levels in the training group decreased (185.21±47.51 mg/dL vs 171.10±37.91 mg/dL), which was statistically significant. But, HDL-C levels in the training group increased (38.20±20.65 mg/dL vs 43.80±7.87 mg/dL), which was statistically significant. Also, there was no significant difference between the two groups in lipid profiles (TC, TG, HDL, LDL) levels, but significant changes were observed in vaspin levels between two groups (Table 2). 

**Vaspin relationships with lipid profiles: **Vaspin correlation with lipid profiles is shown in table 3. Serum vaspin concentrations did not correlate with lipid profiles in pre- and post-exercises.

**Table 1:** Anthropometric and Muscle strength performance characteristics before and after 8 weeks of training programs

**Variables**	**Group**	**Baseline**	**8 weeks**	**P value**
Age (years)	RT	49.22±5.7	-	-
	Control	48.62±7.05	-	
Anthropometric measurement				
Height (cm)	RT	169.24±6.95	-	-
	Control	172.81±6.45	-	
Weight (kg)	RT	82.5±12.4	81.3±21.4	0.87
	Control	79.8±9.2	80.90±10.1	
BMI (kg/m^2^)	RT	28.4±6.9	26.3±4.5 *	0.43
	Control	27.8±4.3	27.4±3.7	
PBF (%)	RT	26.1±3.0	24.1±3.2 *	0.05 *
	Control	25.5±4.4	26.3±8.6	
Muscle strength performance				
1-RM Bench Press (kg)	RT	52.4±8.4	85.4±9.7 *	0.00 **
	Control	50.48±24.7	56.7±4.9	
1-RM Knee Extention (kg)	RT	64.7±12.5	80.3±8.5 *	0.00 **
	Control	52.4±15.9	60.1±7.8	
1-RM latpull (kg)	RT	68.4±16.6	94.8±12.2 *	0.00 **
	Control	50.9±3.5	49.2±9.8 *	
1-RM Triceps Extension (kg)	RT	64.4±10.5	87.3±9.5 *	0.00 **
	Control	65.4±10.9	67.1±3.8	
1-RM Heel Raise (kg)	RT	135.3±23.3	169.3±34.4 *	0.00 **
	Control	122.3±24.9	124.1±24.7 *	
1-RM Arm Curl (kg)	RT	55.3±8.1	85.7±14.5 *	0.00**
	Control	58±2.51	63.5±21.9	
1-RM knee flextion (kg)	RT	55.1±7.2	92.4±10.6 *	0.00 **
	Control	59.8±12.2	59.3±3.1	
1-RM Seated Rowing (kg)	RT	98±10.24	135.3±10.5 *	0.00**
	Control	87±21.8	84.3±18.2 *	

RT: resistance training. Cm: *centimeter*. Kg/m2: kilogram per square metre. ml/kg/min milliliters of oxygen per kilogram of body weight per minute. Kg: kilogram. BMI: Body mass index, PBF: percent body fat.

* Significant difference in compare to Pre values (p≤0.05).

** P≤0.05, significantly different between groups.

**Table 2:** Metabolic parameters, and vaspin levels before and after 8 weeks of training programs

**Variables**	**Group**	**Baseline**	**8 weeks**	**P value**
Vaspin (ng/ml)	RT	330.50 ±82.51	251.62±107.28 *	0.000 **
	Control	344±78.64	436±70.47 *	
TC (mg/dL)	RT	185.21±47.51	171.10±37.91 *	0.65
	Control	180.50±25.78	187.87±28.33	
TG (mg/dL)	RT	180.45±72.44	168.9±45.60	0.08
	Control	175.75±75.24	170.87±32.99	
HDL-C (mg/dL)	RT	38.20±20.65	43.80±7.87 *	0.62
	Control	40.22±15.24	39.89±14.21	
LDL-C (mg/dL)	RT	87.40±34.95	82.60±22.72	0.42
	Control	90.12±14.07	98.50±77.36	

RT: resistance exercise. Mg/dL: milligrams per deciliter. μU/mL: microunits per milliliter. Mmol/mol: millimoles per litre. TC: cholesterol. TG: triglycerides. HDL-c: high density lipoprotein cholesterol. LDL-c: low density lipoprotein cholesterol.

* P<0.05; *Significant difference in compare to Pre values (p≤0.05).

** P≤0.05, significantly different between groups.

**Table 3:** Pearson’s correlation coefficients between plasma vaspin concentration and other parameters at baseline and end of programs

**Variables**	**Baseline**		**After 8 weeks**	
	**r**	**P value**	**r**	**P value**
TC (mg/dL)	0.333	0.252	0.332	0.185
TG (mg/dL)	0.578	0.134	0.258	0.240
HDL-C (mg/dL)	0.154	0.235	0.752	0.235
LDL-C (mg/dL)	0.080	0.380	0.152	0.375

## Discussion

**Vaspin changes: **There have been some controversies concerning the effects of resistance exercise training on vaspin levels and their associations with other metabolic parameters. Vaspin is a new adipocytokine linking adipose tissue related to systemic insulin resistance (2). In Caucasians, Klöting et al. reported that vaspin mRNA expression was not detected in all the study subjects, and was more detectable in diabetic patients than in NGT subjects (8). However, no difference was found in serum vaspin level between the diabetic patients and NGT subjects in other studies (4). Youn et al. reported that physical training for 4 weeks in untrained individuals (men and women) causes increased serum vaspin concentrations with weight loss (9). On the other hand, Oberbach et al. observed significant decrease of serum vaspin concentrations after 4 weeks of training in healthy young men (5). However, the result from the current study is in agreement with the recently reported studies that have found a decrease in vaspin concentration after lifestyle modification in adults (10). To our knowledge, only Oberbach et al. have investigated the response of vaspin after an acute bout of exercise (5). It seems that the differences between the results found in the present study and those reported by Youn et al. may be related to the type of exercise (resistance vs. endurance) and this suggests that vaspin serum concentrations decreased by exercise-induced oxidative stress is confirmed in the present study (9). Also, it seems that it can be due to the difference in the age of participants in the different studies. Different growth stages could affect the levels of growth hormone (GH). It has been reported that GH levels strongly influence vaspin regulation and its circulating levels (11). Knowing that most of our subjects are adult men and large variation in levels of GH is expected, this may explain the large variation of vaspin levels between the two groups. 

Regarding lipid profile changes**, l**ow HDL-C levels, elevation in TG levels have been reported in males with T2D (12). In agreement with previous studies in middle-aged patients with T2D (13, 14), in this study, our training program induced a marked increase in HDL levels in RT group without any significant modification in other variables of the lipid profile. The mechanism by which regular physical exercise increases HDL levels is not known. A 2-year study also showed a slight increase in HDL-cholesterol levels with exercise (15). Those studies that show increased HDL generally involved more rigorous training regimens, although there is some disagreement on this point as well (16, 17). The fact that our study demonstrated an increase in HDL with RT in RT group indicates that our subjects may have been exercising at high intensity. Although there are some suggestions that men with low HDL levels are less likely to respond to training than men with higher HDL levels, our data support this concept (18). However, in consistency with other studies of RT and lipid profiles, we found no effect of RT on cholesterol, TG, LDL, VLDL levels (16). Poirier et al. have reported that changes in lipid profiles levels induced by training are related to changes in fat mass (19). Since it appears that there is a positive association between exercise-induced changes in lipid proﬁles and weight loss, our ﬁndings, along with previous studies, suggest that a greater change in body weight and fat mass may be necessary to have a signiﬁcant effect on lipid proﬁles after RT (20). The current study has some limitations. The first limitation of the current study is that the study population is relatively small; however, the study has sufficient power to detect the influence of RT complications on serum vaspin levels; and the second limitation is the short exercise intervention with no long term follow-up and without dietary and other lifestyles associated with physical activity levels control in exercise training period.

In conclusion, this study showed that RT significantly decreased the levels of vaspin in patients with T2D. However, further studies are needed to investigate the role of vaspin in human physiology and to elucidate the contradictory results regarding the effect(s) of exercise intervention on serum vaspin concentration. 

## Notes

**Funding:** This project was fully sponsored by the Research Center of Payam Noor University of Babol with grant number of (30/3078 Date 2009/04/14).
